# Consistently Increasing Numbers of Online Ratings of Healthcare in England

**DOI:** 10.2196/jmir.2157

**Published:** 2012-06-29

**Authors:** Felix Greaves, Christopher Millett

**Affiliations:** ^1^Department of Primary Care and Public HealthImperial College LondonLondonUnited Kingdom

**Keywords:** online reviews, quality transparency, public reporting

In a recent JMIR paper, Gao and colleagues demonstrated the growing number of internet-based ratings of physicians on a commercially-owned website in the USA [1].

In England, in keeping with our National Health Service, we have a government run website that allows patients to rate and comment on their care online in a similar way, but at the level of healthcare provider organisations rather than individuals. The website is called NHS Choices [2]. Gao suggests that their results demonstrate a positive correlation between online ratings and physician quality. We have similarly demonstrated how better online ratings at the organisational level are associated with better clinical outcomes [3], and patient experience measured by surveys [4] in England.

**Figure 1:**
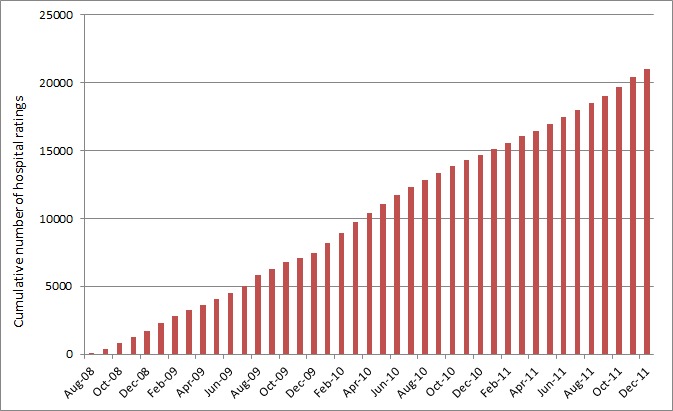
Cumulative number of online ratings of hospitals in England on the NHS Choices website.

In a new analysis to allow comparison with Gao’s results, we looked at the number of ratings of hospitals posted on the NHS Choices website over the period since it started (August 2008) to the end of 2011. There were 20,996 ratings of hospitals over the 40 month period, fewer than in the US. We found a more gradual, linear increase in ratings in England (Figure 1) compared with the accelerating growth in ratings seen on commercial sites in the US [1]. We are not sure why the frequency of ratings is stable in England, but not increasing at the same rate as in the US. This may be because marketing budgets are lower for an English government run service compared to the more commercial advertising approach of US websites, leading to lower awareness of the websites in England. Alternatively, patients in England may be less culturally familiar with the concept of provider choice in healthcare, as the ability to choose between providers has only been introduced relatively recently in the English NHS while it may be a cultural norm in the US. This might result in English patients being less inclined to rate their care. We hope this adds to the work of our American colleagues, and demonstrates that the increasing number of online ratings of healthcare is an international phenomenon, even if England is perhaps at an earlier stage on the curve than the US.
